# Dissemination of a *bla*_NDM − 1_-harboring IncH plasmid associated with concurrent ST30 *Klebsiella pneumoniae* and ST2 *Klebsiella oxytoca* outbreaks in a Chinese neonatal unit

**DOI:** 10.3389/fmicb.2026.1727443

**Published:** 2026-03-06

**Authors:** Dandan Dong, Luze Zou, Zhenzhen Liu, Nan Jia, Yanfei Liu, Hui Zhao, Yuanqi Zhu

**Affiliations:** Department of Clinical Laboratory, The Affiliated Hospital of Qingdao University, Qingdao, China

**Keywords:** BlaNDM-1, *Klebsiella oxytoca*, *Klebsiella pneumoniae*, neonates, outbreak, plasmid

## Abstract

**Objectives:**

Carbapenem-resistant *Klebsiella pneumoniae* (CRKP) and *Klebsiella oxytoca* (CRKO) strains threaten neonatal health. This study investigates concurrent outbreaks in a Chinese neonatal intensive care unit (NICU).

**Methods:**

We characterized 13 clinical isolates (10 CRKP [TJ01–TJ10] and 3 CRKO [TJ11–TJ13]) recovered from preterm infants between September 2013 and January 2014. The whole genome sequencing of strains (CRKP TJ03 and CRKO TJ11) was performed using MiSeq and MinION platforms, while the plasmids pNDM-TJ03 and pNDM-TJ11 were derived from the above-mentioned strains. Antimicrobial susceptibility testing, plasmid conjugal transfer, and other experiments were conducted.

**Results:**

PFGE revealed clonal dissemination of CRKP ST30 and CRKO ST2 strains. This represents both the first documented neonatal outbreak caused by CRKP ST30 globally and the first report of CRKO ST2 in China, demonstrating novel transmission patterns of these high-risk clones in neonatal settings. All isolates carried *bla*_NDM − 1_, *bla*_OXA − 1_, *bla*_DHA − 1_, *qnrB4*, and *aac(6*′*)-Ib-cr* genes. Plasmid analysis identified both as IncH-type, showing high homology with pNDM-MAR. Their structure included contained a conserved IncH backbone region and separate accessory modules that contained five insertion sequences, transposon Tn*6344*, and a multidrug-resistant (MDR) region. The MDR region contained four mobile elements (ΔTn*125*, ΔInRBDHA, ΔTn*1548*, and ΔTn*5393c*) carrying a complement of 10 resistance genes. Conjugation experiments confirmed successful transfer of both plasmids to *Escherichia coli* J53Azi^R^.

**Conclusions:**

This study demonstrates that there may have been intra-strain and inter-species spread of a *bla*_NDM − 1_-harboring IncH plasmid in the NICU. Our findings provide new insights into horizontal transfer of resistance genes mediated by IncH-type plasmids.

## 1 Introduction

*Klebsiella pneumoniae* and *Klebsiella oxytoca* are opportunistic pathogens associated with severe nosocomial infections including pneumonia, urinary tract infections, and bloodstream infections, posing a particularly high risk to neonates in healthcare settings (Gundeslioglu et al., 2024; Minotti et al., 2025). Carbapenem-resistant *K. pneumoniae* (CRKP) and *K. oxytoca* (CRKO) contribute significantly to morbidity and mortality in pediatric population, primarily driven by the acquisition of carbapenemase genes (Montagnani et al., 2016). Among these, New Delhi metallo-β-lactamase (NDM), particularly the *bla*_NDM − 1_ variant, represents a significant public health threat owing to their capacity to confer resistance to nearly all β-lactam antibiotics and its rapid global dissemination (Khan et al., 2017).

Since its initial identification in a *K. pneumoniae* isolate from Sweden with a travel history to India in 2008, *bla*_NDM − 1_ has been reported in over 60 countries and across a wide range of bacterial species, demonstrating substantial potential for cross-border and interspecies transmission (Yong et al., 2009; Yao et al., 2024). The global spread of *bla*_NDM − 1_ is largely attributed to its localization on plasmids, where the gene is typically embedded within mobile genetic elements, especially the composite transposon Tn*125* flanked by the IS*Aba125* element (Poirel et al., 2012). While plasmids belonging to the IncF, IncC, and IncX3 incompatibility groups are recognized as primary vectors driving global dissemination (Wu et al., 2019), other plasmid types, such as IncH plasmids, have increasingly been implicated in regional outbreaks and hospital-acquired transmissions (Bonnin et al., 2020; Chudejova et al., 2021).

Despite advances in understanding NDM epidemiology in adult populations, data focusing specifically on the molecular epidemiology and transmission dynamics of NDM-producing *Klebsiella* strains in neonates remain critically limited (Montagnani et al., 2016). Therefore, this study aimed to investigate a cluster of NDM-producing *Klebsiella* isolates identified in the NICU in China. The objectives were to (1) analyze the molecular epidemiology of 13 clinical isolates (10 CRKP and 3 CRKO) harboring *bla*_NDM − 1_, (2) characterize the genetic architecture of the conjugative plasmids carrying these resistance genes, and (3) elucidate the mechanisms facilitating horizontal gene transfer in this high-risk clinical environment.

## 2 Materials and methods

### 2.1 Bacterial strains, and identification and antimicrobial susceptibility testing

Between September 2013 and January 2014, 10 CRKP (TJ01–TJ10) and 3 CRKO (TJ11–TJ13) isolates were recovered from throat swab specimens of 13 preterm infants admitted to the neonatal intensive care unit (NICU) of a tertiary hospital in China. These neonates had low birth weights (< 2.5 kg), were diagnosed with pneumonia, and their blood cultures were negative. The 13 isolates were identified and subjected to antimicrobial susceptibility testing using a VITEK Compact 2 system (BioMérieux, France). The breakpoints were interpreted using the Clinical and Laboratory Standards Institute guidelines (CLSI, 2025; Schuetz et al., 2025).

### 2.2 Antimicrobial resistance gene screening

The antimicrobial resistance genes, including carbapenem-resistance genes (*bla*_AIM_, *bla*_BIC_, *bla*_DIM_, *bla*_GIM_, *bla*_IMP_, *bla*_KPC_, *bla*_NDM_, *bla*_OXA − 48_, *bla*_SIM_, *bla*_SPM_, and *bla*_VIM_), extended-spectrum β-lactamase genes (*bla*_CTX − M_, *bla*_OXA_, *bla*_SHV_, and *bla*_TEM_), AmpC β -lactamase genes (*bla*_ACC_, *bla*_ACT_, *bla*_BIL_, *bla*_CMY_, *bla*_DHA_, *bla*_FOX_, *bla*_LAT_, *bla*_MIR_, and *bla*_MOX_), and plasmid-mediated quinolone resistance genes (*aac(6*′*)-Ib-cr, qepA, qnrA, qnrB, qnrC, qnrD*, and *qnrS*), were detected by PCR as described previously (Chen et al., 2015). The obtained PCR amplicons were sequenced on an ABI 3730 platform (Applied Biosystems, USA).

### 2.3 Plasmid conjugal transfer and plasmid stability

The 13 clinical isolates (TJ01–TJ13) were used as the donors for the conjugative transfer of the plasmids and the sodium azide-resistant *Escherichia coli* J53Azi^R^ strain was used as the recipient cells. The conjugation experiments were conducted as described previously (Virolle et al., 2020). The plasmid stability of the carbapenemase*-*producing isolates and transconjugants was determined as described previously (Zhu et al., 2016).

### 2.4 Pulsed-field gel electrophoresis (PFGE), S1-nuclease-PFGE and Southern blotting

To analyze the clonal relatedness among the 13 isolates, pulsed-field gel electrophoresis (PFGE) with *Xba*I digestion (TaKaRa, China) was carried out as described previously (Murase et al., 2022). The banding patterns were analyzed visually and with BioNumerics software (version 7.6), and interpreted according to the Tenover criteria (Tenover et al., 1995). The sizes and locations of the *bla*_NDM_-carrying plasmids in the clinical isolates and in their transconjugants were evaluated by S1-PFGE and Southern blotting as described previously (Liu et al., 2024).

### 2.5 Detection of carbapenemase activity

To determine the carbapenemase production of the 13 clinical isolates and their corresponding transconjugants, we used the modified carbapenem inactivation method (mCIM) as described previously (Hu and Li, 2021).

### 2.6 Multilocus sequence typing (MLST)

The seven housekeeping genes (*gapA, infB, mdh, pgi, phoE, rpoB, tonB*) for MLST were amplified by PCR and sequenced from the 13 isolates. High-quality consensus sequences were generated from bidirectional sequencing reads and submitted to the PubMLST database (https://pubmlst.org/) for allele assignment and sequence type (ST) determination, using the Institut Pasteur MLST scheme for *Klebsiella* spp.

### 2.7 Whole-genome sequencing (WGS) and bioinformatic analysis

To elucidate the genomic context of the *bla*_NDM − 1_-harboring plasmids and the phylogenetic background of the outbreak strains, one representative isolate from each of the two concurrent outbreaks was selected for in-depth whole-genome sequencing (WGS). Specifically, CRKP TJ03 (representing the ST30 *K. pneumoniae* outbreak) and CRKO TJ11 (representing the ST2 *K. oxytoca* outbreak) were chosen. The genomic DNAs of the CRKP TJ03 and CRKO TJ11 strains were extracted using a Wizard Genomic DNA Purification Kit (Promega, USA), and sequenced on MiSeq (Illumina, USA) and MinION (Oxford Nanopore, UK) platforms. The short MiSeq reads were trimmed to remove poor quality reads using Trimmomatic, and the clean reads were assembled using Newbler2.9. The long MinION reads were combined with the assembled MiSeq reads and hybrid assembled using SPAdes v3.11.1 (Prjibelski et al., 2020). The hybrid assembly produced several scaffolds and the BLASTN analysis showed that one of the scaffolds was very similar to the reference plasmid pNDM-MAR (Villa et al., 2012). Further bioinformatics analysis using an in-house script confirmed that this scaffold was successfully cyclized. This result was verified by mapping the MiSeq reads to the cyclized scaffold using the CLC Genomics Workbench 9.0 (CLC Bio, Denmark). The consensus sequences of the plasmids obtained from the CLC Genomics Workbench 9.0 were considered to be the complete plasmid sequences. Open reading frames and pseudogenes were predicted using RAST2.0, and annotated using BLASTN and the NCBI Conserved Domains Database (https://www.ncbi.nlm.nih.gov/Structure/cdd/wrpsb.cgi). Resistance genes, mobile elements (insertions, transposons, integrons), and other features were annotated using the Tn Number Registry, ISfinder, INTEGRALL, ResFinder3.2, and PlasmidFinder2.1 online databases (Dong et al., 2019). Comparisons of paired and multiple sequences were performed using BLASTN and MUSCLE 3.8.31, respectively. Gene organization was visualized using Inkscape 0.48.1 (https://inkscape.org/en/).

## 3 Results

### 3.1 Clinical features of the isolates

We isolated 10 CRKP (TJ01–TJ10) and 3 CRKO (TJ11–TJ13) strains from throat swab specimens of 13 neonates. All the neonates were preterm infants (<37 weeks) with low birth weight and pneumonia infection, who had undergone continuous positive airway pressure procedures and had a long hospital stay. Of the 13 neonates, 8 (61.5%) were male and 9 (69.2%) were diagnosed as having neonatal sepsis (Table 1).

**Table 1:** Clinical features of the strains.

**Strains**	**Department**	**Isolated_time**	**Sex**	**Age (days)**	**Weight**	**Gestational_week**	**Specimens**	**Clinical diagnosis**
TJ01	Neonatal unit	2014/1/9	Male	5	1,900	32	Throat swab	Preterm infant, pneumonia infection
TJ02	Neonatal unit	2013/9/21	Male	21	1,800	29	Throat swab	Preterm infant, pneumonia infection
TJ03	Neonatal unit	2013/10/6	Male	4	1,450	29	Throat swab	Preterm infant, pneumonia infection, sepsis
TJ04	Neonatal unit	2013/10/30	Female	9	1,810	35	Throat swab	Preterm infant, pneumonia infection, sepsis
TJ05	Neonatal unit	2013/10/29	Male	18	1,100	26	Throat swab	Preterm infant, pneumonia infection, sepsis
TJ06	Neonatal unit	2013/10/31	Female	31	1,240	28	Throat swab	Preterm infant, pneumonia infection, sepsis
TJ07	Neonatal unit	2013/10/31	Male	2	2,460	36	Throat swab	Preterm infant, pneumonia infection, congenital heart disease
TJ08	Neonatal unit	2013/11/28	Male	27	1,480	31	Throat swab	Preterm infant, pneumonia infection, sepsis
TJ09	Neonatal unit	2013/9/10	Male	7	2,430	36	Throat swab	Preterm infant, pneumonia infection
TJ10	Neonatal unit	2013/9/22	Female	9	2,120	36	Throat swab	Preterm infant, pneumonia infection, sepsis, congenital heart disease
TJ11	Neonatal unit	2013/9/3	Female	5	2,400	36	Throat swab	Preterm infant, pneumonia infection, sepsis
TJ12	Neonatal unit	2013/12/14	Male	8	1,730	31	Throat swab	Preterm infant, pneumonia infection, sepsis, congenital heart disease
TJ13	Neonatal unit	2013/12/26	Female	2	1,550	30	Throat swab	Preterm infant, pneumonia infection, sepsis

### 3.2 Antimicrobial susceptibility testing, detection of carbapenemase activity

The 10 CRKP isolates were highly resistant to ampicillin/sulbactam (SAM), piperacillin/tazobactam (TZP), cefazolin (CZ), cefotetan (CTT), ceftazidime (CAZ), ceftriaxone (CRO), cefepime (FEP), aztreonam (ATM), ertapenem (ETP), imipenem (IPM), nitrofurantoin (NIT), and ciprofloxacin (CIP), and were sensitive to amikacin (AK), gentamicin (CN), tobramycin (TOB), and sulfamethoxazole/trimethoprim (SXT). The three CRKO isolates had the same antibiotic resistance profiles as the 10 CRKP isolates, except for ATM (Table S1).

The modified carbapenem inactivation method (mCIM) was used to detect carbapenemase activity. The results showed that carbapenem inactivation was positive in all 13 clinical isolates and in the 13 corresponding transconjugants.

### 3.3 Detection of antibiotic resistance genes

The PCR screening and PCR amplicon sequencing for antimicrobial resistance genes showed that the 13 clinical isolates carried *bla*_NDM − 1_, *bla*_OXA − 1_, *bla*_DHA − 1_, *qnrB4*, and *aac(6*′*)-Ib-cr* genes, and that the 10 CRKP isolates also carried the *bla*_SHV − 12_ gene.

### 3.4 Molecular typing of the isolates (PFGE and MLST)

**Figure 1:**
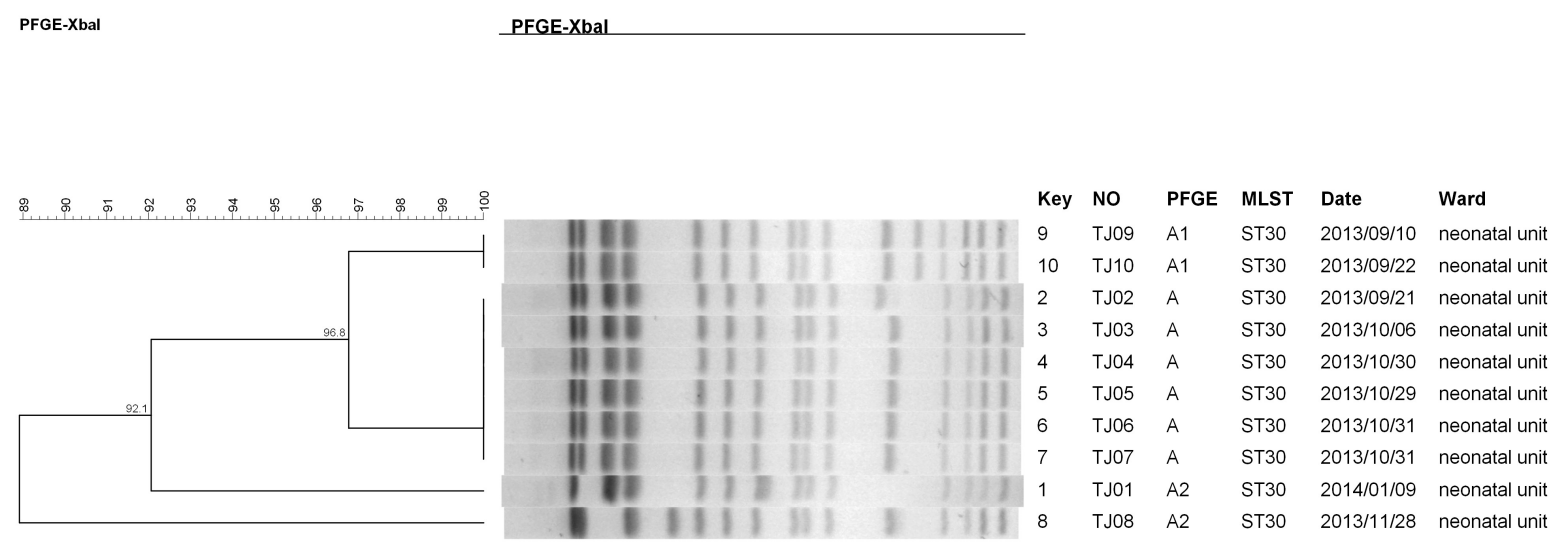
The clonal relatedness of 10 *K. pneumoniae* strains (TJ01–TJ10). The clonal relatedness of strains using pulsed field gel electrophoresis (PFGE) with *Xba*I digestion. the PFGE patterns were then compared by BioNumerics software(version 7.6), and 90% similarity was used as a cutoff to identical PFGE types (pulsotypes).

**Figure 2:**
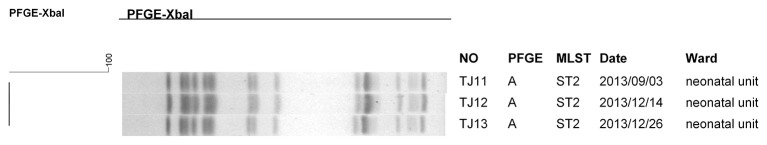
The clonal relatedness of 3 *K. oxytoca* strains (TJ11–13). The clonal relatedness of strains using pulsed field gel electrophoresis (PFGE) with *Xba*I digestion. the PFGE patterns were then compared by BioNumerics software (version 7.6), and 90% similarity was used as a cutoff to identical PFGE types (pulsotypes).

PFGE was performed with the restriction endonuclease *Xba*I to characterize the genetic relatedness among the 13 clinical isolates, and minor differences were observed in their *Xba*I PFGE patterns (Figure S1). Three *Xba*I PFGE patterns were found among the 10 CRKP isolates and were classified according to the Tenover criteria (Tenover et al., 1995). Six of the CRKP isolates (TJ02–TJ07) were indistinguishable and were classified as PFGE pattern type A, and the other four isolates were closely or possibly related and were classified as type A1 (TJ09 and TJ10) and type A2 (TJ01 and TJ08). The Dice coefficients for the 10 CRKP isolates were 92.1%−100% as determined using the BioNumerics software (version 7.6), and all 10 isolates belonged to the ST30 strain (Figures 1, S1). The three CRKO isolates (TJ11–TJ13) had indistinguishable *Xba*I PFGE patterns, the Dice coefficients were 100%, and all three isolates belonged to the ST2 strain (Figures 2, S1). These results suggest that the 10 CRKP isolates as well as the three CRKO isolates were epidemiologically linked.

### 3.5 Plasmid transfer, S1-PFGE and Southern blotting, and plasmid stability

To identify the *bla*_NDM − 1_-carrying plasmids in the 13 clinical isolates, we performed gene transfer experiments by liquid mating with *E. coli* J53Azi^R^ as the recipient cells. The results showed that all 13 isolates transferred the *bla*_NDM − 1_-carrying plasmids to *E. coli* J53Azi^R^, thereby producing 13 transconjugants (TJ01C–TJ13C). Furthermore, the antibiotic resistance patterns of the 13 transconjugant were similar to those of the 13 clinical isolates, except for TZP, FEP, ATM, IPM, and NIT (Table S1).

We performed S1-PFGE and Southern blotting to verify the size and location of the *bla*_NDM_-carrying plasmids in three clinical isolates (TJ03, TJ08, and TJ11) and the corresponding transconjugants (TJ03C, TJ08C, and TJ11C). The S1-PFGE showed that CRKP isolates TJ03 and TJ08 and the corresponding transconjugant TJ03C and TJ08C all carried one plasmid of approximately 280 kb, and that CRKO isolate TJ11 carried two plasmids of approximately 275 kb and 100 kb, whereas its transconjugant TJ11C carried only one plasmid of approximately 275 kb. The Southern blotting confirmed that the *bla*_NDM − 1_ genes were located on plasmids of approximately 280 kb in TJ03, TJ08, TJ03C, and TJ08C, and of approximately 275 kb in TJ11 and TJ11C (Figure S2). The two *bla*_NDM_ -carrying plasmids of approximately 280 kb in CRKP TJ03 and 275 kb in CRKO TJ11 were assigned as pNDM-TJ03 and pNDM-TJ11, respectively.

We selected the CRKP isolate TJ03, which contained pNDM-TJ03, and the CRKO isolate TJ11, which contained pNDM-TJ11, to determinate plasmid stability. The results showed that the two plasmids were stable. After 10 rounds of subculture in MacConkey agar with no added antibiotics, all the randomly selected strains harbored a plasmid identical in size to pNDM-TJ03 and pNDM-TJ11, and carried the *bla*_NDM − 1_ gene as verified by PCR and sequencing.

### 3.6 Characteristics of plasmids pNDM-TJ03 and pNDM-TJ11

**Figure 3:**
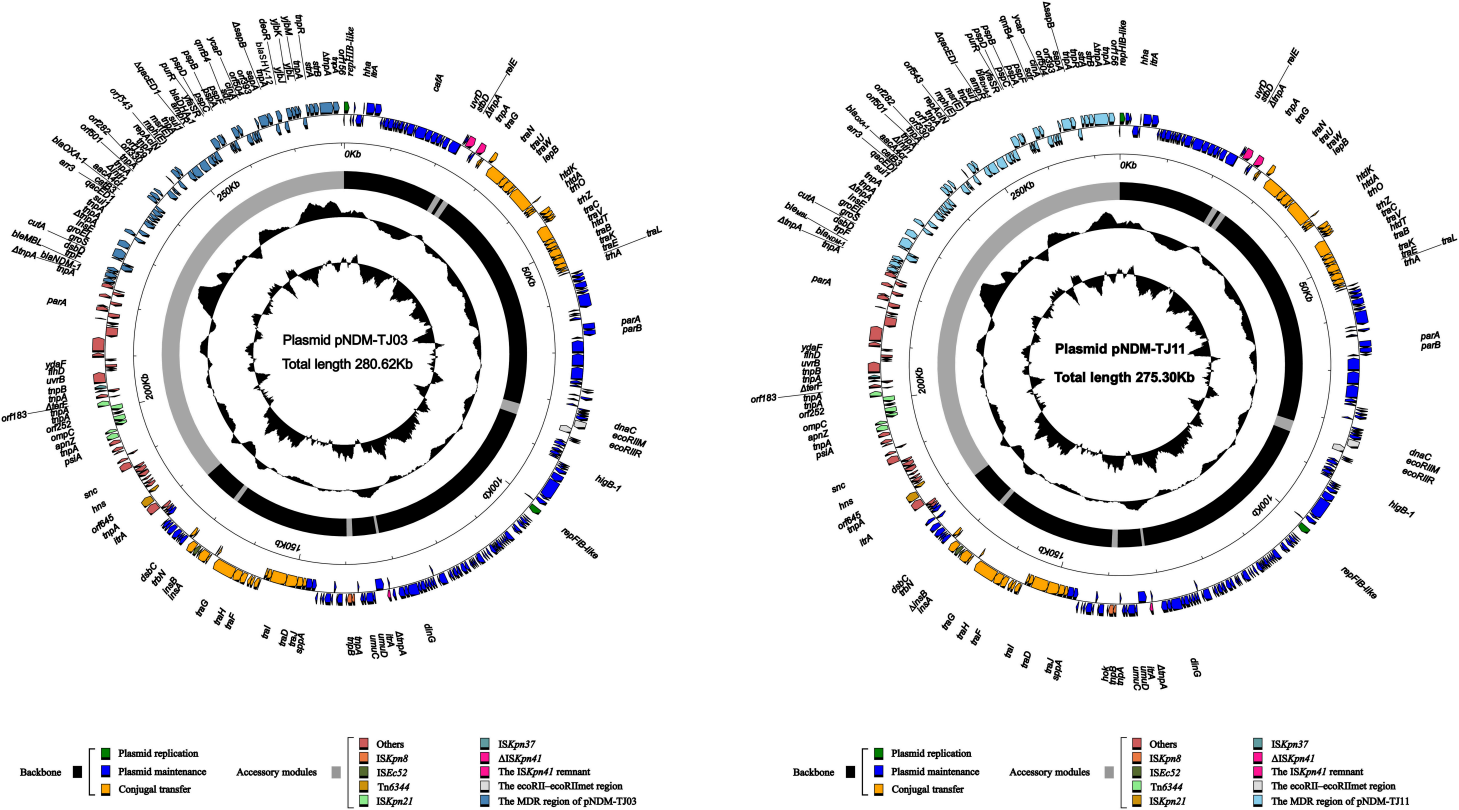
Schematic maps of plasmids pNDM-TJ03 and pNDM-TJ11. Genes are indicated by arrows. The backbone and accessory module regions are highlighted in black and color, respectively. The innermost circle presents GC-skew [(G – C)/(G + C)], with a window size of 500bp and a step size of 20-bp. The next-to-innermost circle shows GC content.

The selected CRKP TJ03 and CRKO TJ11 strains were sequenced on MiSeq and MinION platforms and the complete sequences of plasmids pNDM-TJ03 and pNDM-TJ11, which were present in these strains, were obtained. Plasmid pNDM-TJ03 was 280,625-bp long, with mean GC content of 47%, and pNDM-TJ11 was 275,300-bp long, with mean GC content of 46% (Table 2). On the basis of the replicon, pNDM-TJ03 and pNDM-TJ11 were assigned to the IncH incompatibility group. Linear genomic comparisons were conducted among pNDM-TJ03, pNDM-TJ11, and pNDM-MAR. The results showed that pNDM-TJ03 and pNDM-TJ11 shared high sequence homology with pNDM-MAR (Villa et al., 2012), with >72% query coverage and >99% identity (Figure S3). The molecular structures of pNDM-TJ03 and pNDM-TJ11 contained conserved IncH backbone regions and separate accessory modules that contained five insertion sequences (ΔIS*Kpn41*, IS*Kpn8*, IS*Ec52*, IS*Kpn21*, and IS*Kpn37*), a transposon Tn*6344*, and a multidrug-resistant (MDR) region (Figures 3, S3).

**Table 2:** Major features of plasmids in this work.

**Category**	IncH plasmids		
	**pNDM-TJ03**	**pNDM-TJ11**	**pNDM-MAR**
Accession number	MG845201 (this study)	MG845200 (this study)	NC_016980
Strain	*K. pneumoniae*	*K. oxytoca*	*K. pneumoniae*
Source	Throat swab	Throat swab	Urine
Country	China	China	Morocco
Total length (bp)	280,625	275,300	267,242
Total number of ORFs	412	398	391
Mean G + C content, %	47%	46%	47%
Resistance genes	*bla*_NDM − 1_, *sul1, qacEDl, arr3, catB3*, *bla*_OXA − 1_, *aacA4cr, mph*(E), *bla*_DHA − 1_, *qnrB4*, *bla*_SHV − 12_, *strA, strB*	*bla*_NDM − 1_, *sul1, qacEDl, arr3, catB3*, *bla*_OXA − 1_, *aacA4cr, mph*(E), *bla*_DHA − 1_, *qnrB4, strA, strB*	*qnrB1*, *bla*_NDM − 1_, *aacA4cr*, *bla*_OXA − 1_, *catB4*, *bla*_CTX − M−15_, *catA*

The backbone regions of the three plasmids were further divided into three parts comprising (i) the replication genes (*repA*) and its iterons), (ii) the conjugal transfer genes (*tra, trb, trh*, and *htd*), and (iii) the plasmid maintenance genes (*stbD, parABC, umuCD*, and *dsbC*). Pairwise comparison analysis of the backbones of the three plasmids showed that they shared >99% nucleotide identity across >95% of their sequences, indicating that the backbones were relatively conserved. However, there were two major differences among their backbones: (i) the copy numbers of the 10-bp tandem repeat (AGGGCAGGGC) in the *parC* gene (centromere, binding sites for *parB*) varied (5 in pNDM-MAR, 6 in pNDM-TJ03, and 7 in pNDM-TJ11); and (ii) compared with the conjugal transfer region in pNDM-MAR (Villa et al., 2012), conjugal transfer region 2 in pNDM-TJ03 and pNDM-TJ11 was truncated by insertion of IS*Ec52* (Figures 3, S3).

**Figure 4:**
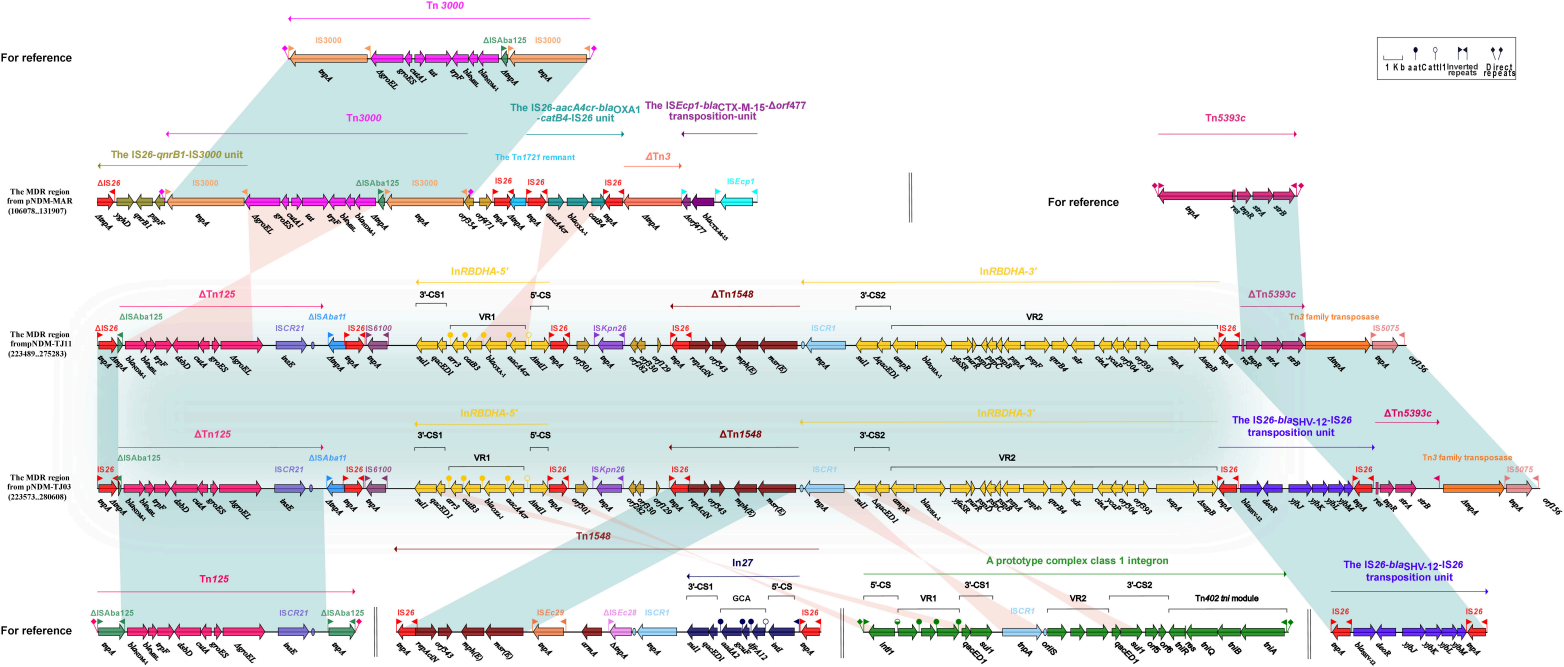
The MDR region harboring *bla*_NDM − 1_ of plasmids (pNDM-TJ03 and pNDM-TJ11) and comparison with the reference plasmid pNDM-MAR. The accession number of Tn*125*, InRBDHA, Tn*1548*, the IS*26*-*bla*_SHV − 12_-IS*26* transposition unit, and Tn*5393c* for reference are NC_019268, AJ971343, AF550415, CP003684, and AF262622, respectively. Genes are denoted by arrows. Mobile elements, genes, and other features are colored based on function classification. Numbers in parentheses denote GenBank numbers and the nucleotide positions within the corresponding plasmids. Shading regions show shared DNA regions of homology (>95% nucleotide identity).

The *bla*_NDM − 1_ gene was located at the MDR region of pNDM-TJ03 and pNDM-TJ11, which contained mobile elements ΔTn*125*, ΔInRBDHA, ΔTn*1548*, and ΔTn*5393c* that carried drug resistance genes. In pNDM-TJ03 and pNDM-TJ11, ΔTn*125* had undergone truncation of upstream and deletion of downstream IS*Aba125*, and the reverse ΔInRBDHA was truncated because of the insertion of a IS*26*–IS*Kpn26*–ΔTn*1548* fragment upstream of IS*CR1*. Additionally, ΔTn*1548* contained IS*26, repAciN, orf543, mph*(E), and *msr*(E) in pNDM-TJ03 and pNDM-TJ11. However, compared with the MDR region of plasmid pNDM-TJ11, the IS*26*–*bla*_SHV − 12_-IS*26* transposition unit in the MDR region of plasmid pNDM-TJ03 was inserted between integron InRBDHA-3′ and transposon ΔTn*5393c*. Tn*5393c* contained IRL (inverted repeat left), *tnpR, res, tnpA, strA, strB*, and IRR (inverted repeat right) in plasmid pRAS2 from the fish pathogen *Aeromonas salmonicida* subsp. *salmonicida*, bordered by 5-bp direct repeats (L'Abée-Lund and Sørum, 2000), whereas ΔTn*5393c* in pNDM-TJ03 and pNDM-TJ11 contained *res, tnpR, strA, strB*, and IRR (Figure 4).

## 4 Discussion

NDM-producing Enterobacteriaceae are notable for their rapid dissemination. The metallo-β-lactamase NDM-1 efficiently hydrolyzes all classes of β-lactam antibiotics except ATM (aztreonam), and is associated with multiple determinants that enable bacteria to become resistant to other antibiotic classes. Therefore, treatment options for NDM-producing Enterobacteriaceae infections are limited. Furthermore, epidemiological surveillance of NDM-1 producers is important for clinical infection control. In this study, we reported ten CRKP and three CRKO isolates from Chinese neonates who were hospitalized between September 2013 and January 2014.

Importantly, the strains were collected from 13 infants with birth weight <2.5 kg diagnosed with pneumonia. Previous studies have shown that overall mortality of neonates was associated with birth weight, premature small for gestational age, and infection complications (Nour et al., 2017), and that mechanical ventilation and long hospital stays increased the rate of hospital-acquired infections (Tess et al., 1993). Moreover, neonatal sepsis are the leading causes of morbidity and mortality in pediatrics (Kim et al., 2020). The Global Burden of Disease Study 2016 indicated that neonatal sepsis and other neonatal infections ranked third among the causes of neonatal deaths (Tess et al., 1993; Moraga and GBD 2016 Causes of Death Collaborators, 2017). The 13 newborns in our study recovered through effective treatment, although nine of them were diagnosed with pneumonia and sepsis.

Evaluation of DNA correlation, which was performed according to the Tenover criteria (Tenover et al., 1995), showed that the 10 CRKP and 3 CRKO strains were indistinguishable or closely or possibly related. The 13 newborns were hospitalized in the same unit and became infected within the same 3-month period. Furthermore, the MLST analysis revealed that the 10 CRKP and 3 CRKO isolates belonged to the ST30 and ST2 strains, respectively. NDM-1-producing *K. pneumoniae* strains, including globally distributed lineages like ST11, ST14, ST15, and ST147, have been identified across multiple continents (Wu et al., 2019). In China, ST11 is the most reported type (Zhang et al., 2018). Notably, the ST30 lineage has been detected in both China and the United States (Dong et al., 2015; Rojas et al., 2017), with one study reporting a foodborne ST30 isolate harboring *bla*_NDM − 1_ that may have originated from food handlers (Khalifa et al., 2020). However, despite these sporadic reports, a clonal outbreak of *K. pneumoniae* ST30 had not been documented to date in newborns. *K. oxytoca* ST2 has been documented in six countries, namely Denmark, Germany, Spain, Switzerland, the United Kingdom, and the United States (Izdebski et al., 2015). However, it has not been previously reported in China. Therefore, our data suggest there was horizontal clonal spread of the CRKP ST30 and CRKO ST2 strains among the neonates in the NICU, demonstrating novel transmission patterns of these high-risk clones in neonatal settings.

The *bla*_NDM − 1_ gene was first identified in a *K. pneumoniae* strain isolated from a New Delhi hospital in India (Yong et al., 2009). Among the known NDM variants, NDM-1 exhibits the broadest host range, having been detected across bacterial species belonging to 11 distinct families (Wu et al., 2019). Consequently, *K. pneumoniae* serves as the primary reservoir for *bla*_NDM_ within the Enterobacteriaceae family, accounting for slightly more than half of all clinical isolates (Wu et al., 2019). The *bla*_NDM − 1_ gene has been widely disseminated across 62 countries, with a notably higher prevalence observed in coastal regions (Yao et al., 2024). The rapid global dissemination of *bla*_NDM − 1_ is primarily facilitated by its localization on conjugative plasmids, often embedded within mobile genetic elements such as the IS*Aba125*-based composite transposon Tn*125*, which was initially characterized in plasmid pNDM-BJ01 from an *Acinetobacter lwoffii* strain (Poirel et al., 2012; Chen et al., 2015). The Tn*125* transposon comprised IS*Aba125*, *bla*_NDM − 1_, *ble*_MBL_, t*rpF, dsbD, cutA, groES, groEL*, and IS*CR21*, bounded by 3-bp direct repeats (target site duplication signals for transposition). Compared with Tn*125*, ΔTn*125* is a reversed fragment from Δ*groEL* to ΔIS*Aba125* in pNDM-MAR (Villa et al., 2012), but it is in a large composite transposon Tn*3000*.

We found that the *bla*_NDM − 1_ gene was located at the MDR region of pNDM-TJ03 and pNDM-TJ11 that carried drug resistance genes. The mobile elements in the MDR region included ΔTn*125*, ΔInRBDHA, ΔTn*1548*, and ΔTn*5393c*. In pNDM-TJ03 and pNDM-TJ11, ΔTn*125* had undergone truncation of upstream and deletion of downstream IS*Aba125*. A complex class 1 integron InRBDHA has been studied in detail in plasmid pRBDHA from *K. pneumoniae*. The plasmid contains a5′-conserved segment 5′-CS: *intI1–attI1*), variable region 1 (VR1: *aacA*4*c*−−*bla*_OXA − 1_-*catB3*–*arr3*), a 3′-conserved segment 1(3′-CS1: *qacED1–sul1*), IS*CR1* (common region), variable region 2 (VR2: the *bla*_DHA − 1_ and *sap*–*qnrB4*–*psp* regions), and a 3′-conserved segment 2 (3′-CS2: Δ*qacED–sul1*; Verdet et al., 2006). VR1 was recovered from integron In37 of an *E. coli* strain in Shanghai, China (Wang et al., 2003). The resistance gene *bla*_DHA − 1_ of VR2 was obtained from the *Morganella morganii* genome, and then the *sap*–*qnrB4*–*psp* region was integrated into InRBDHA by IS*CR1*-mediated transposition (Yim et al., 2013). In our study, the reverse ΔInRBDHA was truncated because of the insertion of a IS*26*–IS*Kpn26*–ΔTn*1548* fragment upstream of IS*CR1*. Tn*1548* is an IS*26*-bordered composite transposon from plasmid pCTX-M3 in *Citrobacter freundii* (Zacharczuk et al., 2011) that had the modular structure IS*26*–In*518*–IS*CR1*–ΔIS*Ec28*–*armA*–IS*Ec29–msr*(E)–*mph*(E)–IS*26*. The *msr*(E)–*mph*(E) operon encodes macrolide efflux protein and macrolide2′-phosphotransferase, respectively, and accounts for macrolide resistance. In pNDM-TJ03 and pNDM-TJ11, ΔTn*1548* contained IS*26, repAciN, orf543, mph*(E), and *msr*(E). However, compared with the MDR region of pNDM-TJ11, the IS*26*–*bla*_SHV − 12_-IS*26* transposition unit in the MDR region of pNDM-TJ03 was inserted between integron InRBDHA-3′ and transposon ΔTn*5393c*. Tn*5393* has three isoforms, Tn*5393a* (Harmer, 2021), Tn*5393b* (Sundin and Bender, 1995), and Tn*5393c* (Selvaraj et al., 2022), that have different insertion sequences. Tn*5393c* contained IRL, *tnpR, res, tnpA, strA, strB*, and IRR in plasmid pRAS2 from the fish pathogen *Aeromonas salmonicida* subsp. *salmonicida*, bordered by 5-bp direct repeats (Selvaraj et al., 2022). In contrast, ΔTn*5393c* from pNDM-TJ03 and pNDM-TJ11 contained *res, tnpR, strA, strB*, and IRR.

Horizontal gene transfer is an essential component of bacterial evolution. In this study, we carried out liquid mating experiments with *E. coli* J53Azi^R^. The results showed that pNDM-TJ03 and pNDM-TJ11 can be transferred, and this finding was confirmed by S1-PFGE and Southern blotting although the two plasmids were stable in the plasmid stability experiments. Therefore, on the basis of the comparative analysis of the complete plasmid sequencing and conjugation results, we presume there was intra-strain and inter-species spread of an IncH plasmid that harbored *bla*_NDM − 1_ in the NICU.

## 5 Conclusions

This study provides critical insights into the molecular epidemiology of carbapenem-resistant *Klebsiella* species in neonatal settings. We report the co-occurrence of two distinct dissemination mechanisms: (i) clonal expansion of high-risk lineages (*K. pneumoniae* ST30 and *K. oxytoca* ST2), representing the first documented neonatal outbreak of CRKP ST30 globally and the initial identification of CRKO ST2 in China; and (ii) horizontal transfer of *bla*_NDM − 1_-bearing IncH plasmids (pNDM-TJ03 and pNDM-TJ11) carrying multiple additional resistance determinants (*bla*_OXA − 1_, *bla*_DHA − 1_, *qnrB4*, and *aac(6*′*)-Ib-cr*).

## Data Availability

The datasets presented in this study can be found in online repositories. The names of the repository/repositories and accession number(s) can be found below: https://www.ncbi.nlm.nih.gov/genbank/, MG845201 https://www.ncbi.nlm.nih.gov/genbank/, MG845200.

## Supplementary material

Figure S1 The clonal relatedness of strains using pulsed field gel electrophoresis (PFGE). The clonal relatedness of strains using pulsed field gel electrophoresis (PFGE) with XbaI digestion. M, lambda ladder PFG marker; Lane 1–10, *K. pneumoniae* (TJ01–10) strains; Lane 11–13, *K. oxytoca* (TJ11–13) strains.

Figure S2 The location analysis of *bla*_NDM − 1_. **(a)** S1 nuclease pulsed-field gel electrophoresis (PFGE) from *K. pneumoniae, K. oxytoca* strains and their transconjugants (Lan1-6:TJ03, TJ03C, TJ08, TJ08C, TJ11, TJ11C), Lane M: marker lambda ladder PFG marker (New England Biolabs, USA); **(b)** Southern hybridization from *K. pneumoniae* strains and transconjugants using a *bla*_NDM − 1_-specific probe (Lan1–4: TJ03, TJ03C, TJ08, TJ08C); **(c)** Southern hybridization from *K. oxytoca* strain and transconjugants using a *bla*_NDM − 1_-specific probe (Lan5–6: TJ11, TJ11C).

Figure S3 Linear comparison of plasmids (pNDM-TJ03 and pNDM-TJ11) and the reference plasmid pNDM-MAR. A linear comparison was carried out for the complete DNA sequences of plasmids pNDM-TJ03 and pNDM-TJ11 (this study) and pNDM-MAR (Accession no. JN420336). Genes are denoted by arrows. Genes, mobile elements and other features are colored based on function classification. Shading indicates regions of homology (>95% nucleotide identity). MDR, multidrug-resistant.

Table S1 Antimicrobial susceptibility profiles.
