# Supplementary figures and images for: Dissemination of a blaNDM − 1-harboring IncH plasmid associated with concurrent ST30 Klebsiella pneumoniae and ST2 Klebsiella oxytoca outbreaks in a Chinese neonatal unit
Source: Front Microbiol. 2026 Mar 6;17:1727443. doi: 10.3389/fmicb.2026.1727443 (PMC13003566; doi:10.3389/fmicb.2026.1727443)

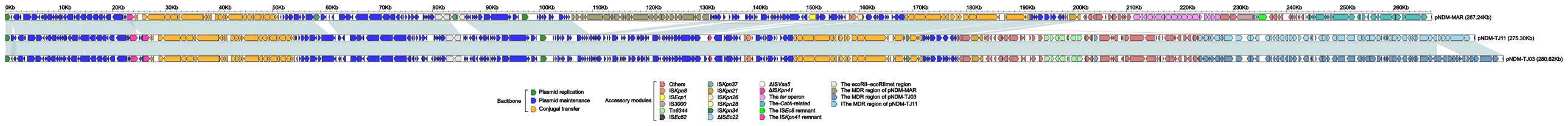

Supplement: Figure S1 — The clonal relatedness of strains using pulsed field gel electrophoresis (PFGE). The clonal relatedness of strains using pulsed field gel electrophoresis (PFGE) with XbaI digestion. M, lambda ladder PFG marker; Lane 1–10, K. pneumoniae (TJ01–10) strains; Lane 11–13, K. oxytoca (TJ11–13) strains. [file Data_Sheet_1.zip › Figure_S3.TIFF]
