# Supplementary material for: Dissemination of a blaNDM − 1-harboring IncH plasmid associated with concurrent ST30 Klebsiella pneumoniae and ST2 Klebsiella oxytoca outbreaks in a Chinese neonatal unit
Source: Front Microbiol. 2026 Mar 6;17:1727443. doi: 10.3389/fmicb.2026.1727443 (PMC13003566; doi:10.3389/fmicb.2026.1727443)
Supplement: Table S1 — Antimicrobial susceptibility profiles. [file Table_1.docx]

Supplementary Material

**Table S1. Antimicrobial susceptibility profiles.**

| **Strains** | **MIC (mg/L)/antimicrobial susceptibility** | | | | | | | | | | | | | | | | | |
| --- | --- | --- | --- | --- | --- | --- | --- | --- | --- | --- | --- | --- | --- | --- | --- | --- | --- | --- |
|  | AMP | SAM | TZP | CZ | CTT | CAZ | CRO | FEP | ATM | ETP | IPM | AK | CN | TOB | CIP | LE | NIT | SXT |
| TJ01 | ≥32 | ≥32 | ≥128 | ≥64 | ≥64 | ≥64 | ≥64 | ≥64 | ≥64 | ≥8 | ≥16 | ≤2 | ≤1 | 4 | 1 | 1 | 128 | ≤20 |
| TJ01C | ≥32 | ≥32 | 64 | ≥64 | ≥64 | ≥64 | ≥64 | 8 | ≥64 | ≥8 | 8 | ≤2 | ≤1 | ≤1 | ≤0.25 | ≤0.25 | ≤16 | ≤20 |
| TJ02 | ≥32 | ≥32 | ≥128 | ≥64 | ≥64 | ≥64 | ≥64 | ≥64 | ≥64 | ≥8 | ≥16 | ≤2 | ≤1 | 8 | 1 | 1 | 128 | ≤20 |
| TJ02C | ≥32 | ≥32 | 64 | ≥64 | ≥64 | ≥64 | ≥64 | 8 | 16 | ≥8 | 8 | ≤2 | ≤1 | ≤1 | ≤0.25 | 0.5 | ≤16 | ≤20 |
| TJ03 | ≥32 | ≥32 | ≥128 | ≥64 | ≥64 | ≥64 | ≥64 | 32 | ≥64 | ≥8 | ≥16 | ≤2 | ≤1 | 4 | 1 | 1 | 128 | ≤20 |
| TJ03C | ≥32 | ≥32 | 64 | ≥64 | ≥64 | ≥64 | ≥64 | 16 | ≥64 | ≥8 | ≥16 | ≤2 | ≤1 | ≤1 | 0.5 | 0.5 | ≤16 | ≤20 |
| TJ04 | ≥32 | ≥32 | ≥128 | ≥64 | ≥64 | ≥64 | ≥64 | 32 | ≥64 | ≥8 | 8 | ≤2 | ≤1 | 4 | 1 | 1 | 128 | ≤20 |
| TJ04C | ≥32 | ≥32 | 64 | ≥64 | ≥64 | ≥64 | ≥64 | 8 | 16 | ≥8 | 8 | ≤2 | ≤1 | ≤1 | ≤0.25 | 0.5 | 32 | ≤20 |
| TJ05 | ≥32 | ≥32 | ≥128 | ≥64 | ≥64 | ≥64 | ≥64 | 32 | ≥64 | ≥8 | ≥16 | ≤2 | ≤1 | 8 | 1 | 1 | 128 | ≤20 |
| TJ05C | ≥32 | ≥32 | 64 | ≥64 | ≥64 | ≥64 | ≥64 | 32 | ≥64 | ≥8 | 8 | ≤2 | ≤1 | ≤1 | ≤0.25 | 0.5 | ≤16 | ≤20 |
| TJ06 | ≥32 | ≥32 | ≥128 | ≥64 | ≥64 | ≥64 | ≥64 | 32 | ≥64 | ≥8 | ≥16 | ≤2 | ≤1 | 4 | 1 | 1 | 128 | ≤20 |
| TJ06C | ≥32 | ≥32 | 64 | ≥64 | ≥64 | ≥64 | ≥64 | 8 | 16 | ≥8 | 8 | ≤2 | ≤1 | ≤1 | ≤0.25 | 0.5 | ≤16 | ≤20 |
| TJ07 | ≥32 | ≥32 | ≥128 | ≥64 | ≥64 | ≥64 | ≥64 | 32 | ≥64 | ≥8 | ≥16 | ≤2 | ≤1 | 4 | 1 | 1 | 128 | ≤20 |
| TJ07C | ≥32 | ≥32 | 64 | ≥64 | ≥64 | ≥64 | ≥64 | 8 | 16 | ≥8 | 8 | ≤2 | ≤1 | ≤1 | ≤0.25 | ≤0.25 | ≤16 | ≤20 |
| TJ08 | ≥32 | ≥32 | ≥128 | ≥64 | ≥64 | ≥64 | ≥64 | 32 | ≥64 | ≥8 | 8 | ≤2 | ≤1 | 4 | 1 | 1 | 128 | ≤20 |
| TJ08C | ≥32 | ≥32 | 64 | ≥64 | ≥64 | ≥64 | ≥64 | 16 | ≥64 | ≥8 | 8 | ≤2 | ≤1 | 2 | 0.5 | 0.5 | ≤16 | ≤20 |
| TJ09 | ≥32 | ≥32 | ≥128 | ≥64 | ≥64 | ≥64 | ≥64 | 32 | ≥64 | ≥8 | ≥16 | ≤2 | ≤1 | 4 | 1 | 1 | 128 | ≤20 |
| TJ09C | ≥32 | ≥32 | 64 | ≥64 | ≥64 | ≥64 | ≥64 | 16 | 32 | ≥8 | ≥16 | ≤2 | ≤1 | 2 | 0.5 | 0.5 | ≤16 | ≤20 |
| TJ010 | ≥32 | ≥32 | ≥128 | ≥64 | ≥64 | ≥64 | ≥64 | 32 | ≥64 | ≥8 | ≥16 | ≤2 | ≤1 | 4 | 1 | 1 | 128 | ≤20 |
| TJ10C | ≥32 | ≥32 | 64 | ≥64 | ≥64 | ≥64 | ≥64 | 8 | 16 | ≥8 | 8 | ≤2 | ≤1 | ≤1 | ≤0.25 | ≤0.25 | ≤16 | ≤20 |
| TJ11 | ≥32 | ≥32 | ≥128 | ≥64 | ≥64 | ≥64 | ≥64 | 32 | 2 | ≥8 | ≥16 | ≤2 | ≤1 | 8 | 1 | 1 | 32 | ≤20 |
| TJ11C | ≥32 | ≥32 | 64 | ≥64 | ≥64 | ≥64 | ≥64 | 8 | ≤1 | ≥8 | 8 | ≤2 | ≤1 | ≤1 | ≤0.25 | 0.5 | ≤16 | ≤20 |
| TJ12 | ≥32 | ≥32 | ≥128 | ≥64 | ≥64 | ≥64 | ≥64 | ≥64 | 2 | ≥8 | ≥16 | ≤2 | ≤1 | 8 | 1 | 1 | 64 | ≤20 |
| TJ12C | ≥32 | ≥32 | 64 | ≥64 | 16 | ≥64 | ≥64 | 4 | ≤1 | ≥8 | 8 | ≤2 | ≤1 | ≤1 | ≤0.25 | ≤0.25 | ≤16 | ≤20 |
| TJ13 | ≥32 | ≥32 | ≥128 | ≥64 | ≥64 | ≥64 | ≥64 | 16 | 2 | ≥8 | ≥16 | ≤2 | ≤1 | 8 | 1 | 1 | 32 | ≤20 |
| TJ13C | ≥32 | ≥32 | 64 | ≥64 | ≥64 | ≥64 | ≥64 | 8 | ≤1 | ≥8 | 8 | ≤2 | ≤1 | ≤1 | ≤0.25 | 0.5 | ≤16 | ≤20 |
| J53AziR | 8 | 4 | ≤4 | ≤4 | ≤4 | ≤1 | ≤1 | ≤1 | ≤1 | ≤0.5 | ≤1 | ≤2 | ≤1 | ≤1 | ≤0.25 | ≤0.25 | ≤16 | ≤20 |

*AMP (Ampicillin), SAM (Ampicillin/Sulbactam), TZP (Piperacillin/Tazobactam), CZ (Ceftazolin), CTT (Ceftetan), CAZ (Ceftazidime), CRO (Ceftriaxone), FEP (Cefepime), ATM (Aztreonam), ETP (Ertapenem), IPM (Imipenem), AK (Amikacin), CN (Gentamicin), TOB (Tobramycin), CIP (Ciprofloxacin), LE (Levofloxacin), NIT (Nitrofurantoin), SXT (Sulfamethoxazole/Trimethoprim). The results of *Klebsiella pneumoniae* (TJ01~10), *Klebsiella oxytoca* (TJ11~13), and their transconjugants (TJ01C~13C) strains were interpreted by the clinical and laboratory standards institute guidelines (CLSI, 2020) (S=sensitive; R=resistant; I= intermediately resistant.
